# Characteristics of a Novel Manganese Superoxide Dismutase of a Hadal Sea Cucumber (*Paelopatides* sp.) from the Mariana Trench

**DOI:** 10.3390/md17020084

**Published:** 2019-02-01

**Authors:** Yanan Li, Xue Kong, Haibin Zhang

**Affiliations:** 1 Institute of Deep-Sea Science and Engineering, Chinese Academy of Sciences, Sanya 572000, China; liyn@idsse.ac.cn (Y.L.); kongx@sidsse.ac.cn (X.K.); 2 College of Earth and Planetary Sciences, University of Chinese Academy of Sciences, Beijing 100039, China

**Keywords:** expression, purification, deep-sea enzyme, pCold vector

## Abstract

A novel, cold-adapted, and acid-base stable manganese superoxide dismutase (Ps-Mn-SOD) was cloned from hadal sea cucumber *Paelopatides* sp. The dimeric recombinant enzyme exhibited approximately 60 kDa in molecular weight, expressed activity from 0 °C to 70 °C with an optimal temperature of 0 °C, and resisted wide pH values from 2.2–13.0 with optimal activity (> 70%) at pH 5.0–12.0. The *K*m and *V*max of Ps-Mn-SOD were 0.0329 ± 0.0040 mM and 9112 ± 248 U/mg, respectively. At tested conditions, Ps-Mn-SOD was relatively stable in divalent metal ion and other chemicals, such as β-mercaptoethanol, dithiothreitol, Tween 20, Triton X-100, and Chaps. Furthermore, the enzyme showed striking stability in 5 M urea or 4 M guanidine hydrochloride, resisted digestion by proteases, and tolerated a high hydrostatic pressure of 100 MPa. The resistance of Ps-Mn-SOD against low temperature, extreme acidity and alkalinity, chemicals, proteases, and high pressure make it a potential candidate in biopharmaceutical and nutraceutical fields.

## 1. Introduction

Reactive oxygen species (ROS) are necessary for various physiological functions, such as signaling pathways and immune responses; the mass accumulation of ROS will damage bio-macromolecules, leading to cell death and various diseases [1,2]. Superoxide dismutases (SODs, EC 1.15.1.1) are one of the most important antioxidant enzymes that clear ROS by converting them into oxygen and hydrogen peroxide. According to the different metal cofactors, several types, such as Cu,Zn-SOD, Mn-SOD, Fe-SOD, cambialistic SOD (activated with either Fe or Mn), Ni-SOD, and Fe,Zn-SOD, have been reported in many species [3,4,5,6,7].

Studies have shown that SODs are related to immune reactions in invertebrates, as exemplified by bacterial and viral invasion [4,8], environmental pollution [9,10], and temperature stimulation [11]. Recently, Xie et al. indicates that antioxidant is related to the deep-sea environmental adaptability [12]. On the other hand, point mutations and activity loss of SODs lead to serious diseases and death in vertebrates. For example, the mice model of mitochondria SOD-deficiency is characterized by neurodegeneration, myocardial injury, and perinatal death [13,14]. A strong link is observed between Alzheimer’s disease, tumor, amyotrophic lateral sclerosis, and SODs [15,16]. Hence, the physiological significance of SODs allows their application in the therapeutic and nutraceutical fields. To date, SODs have been reported to exhibit positive effects on inflammatory diseases, arthritis tumor, and promotion [17,18,19]. An orally effective form of SOD (glisodin) has been developed by Isocell Pharma, and it showed cosmetic and health benefits in human subjects [20,21]. Producing SOD using engineered bacteria is one of the most promising methods to obtain high yield and inexpensive SODs for application. Therefore, the development of SODs with remarkable characteristics is particularly urgent.

Sea cucumbers are highly important commercial sea foods owing to their high nutritional value, and they are distributed from shallow water to the deep sea [22]. Although deep sea is an extremely low-temperature and high hydrostatic-pressured environment for most living organisms, holothurians dominate benthic megafaunal communities in hadal trenches and form “the kingdom of Holothuroidea” when food is abundant [23]. Extreme environments, such as the deep sea, are ideal for the development of new enzymes; numerous novel enzymes with unique activities, such as proteases and lipases, have been identified from the deep sea [24,25]. Considering the promising applications of SODs in therapeutic and nutraceutical fields, relationship with the adaptability of the deep-sea environment and limited studies in extreme organisms, especially in hadal sea cucumbers, we report a novel manganese superoxide dismutase from hadal sea cucumber *Paelopatides* sp. (Ps-Mn-SOD), which inhabits a depth of 6500 m in the Mariana Trench, analyzed its biochemical characteristics, and evaluated its stability for potential use in the food and preliminarily nutraceutical fields.

## 2. Results

### 2.1. Sequence Characteristics

**Figure 1:**
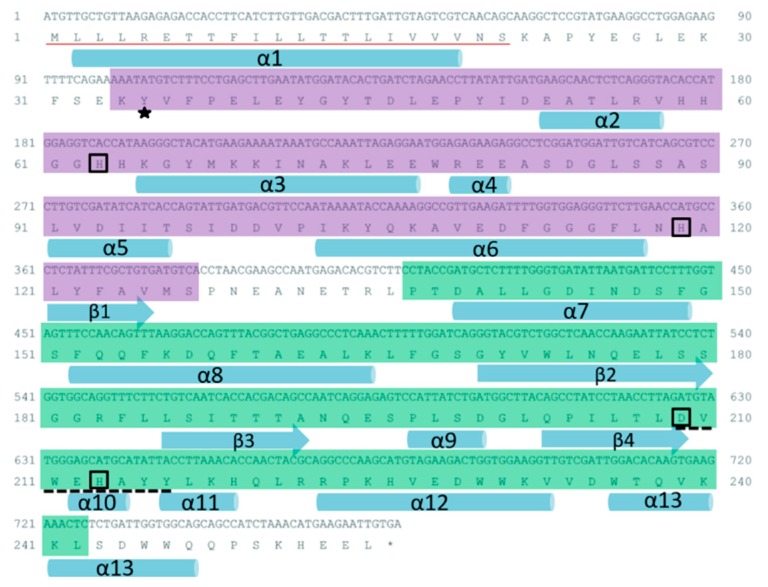
Nucleotide and corresponding amino acid sequences of Ps-Mn-SOD. The signal peptide is drawn with a red line. The signature sequence DVWEHAYY is underlined with dotted line. N- and C-terminal domains are marked with purple and green shades, respectively. Four conserved amino acid residues for manganese coordination are boxed. Asterisk points to the highly conserved Tyr-35 residue. Cylinders and arrows represent helices and strands, respectively.

The ORF of Ps-Mn-SOD is 768 bp long, encoding 255 amino acids. A signal peptide was detected at the N-terminal of deduced amino acid sequence. The N- and C-terminal domains spanned from Lys-34 to Ser-127 and Pro-137 to Leu-242, respectively. Four conserved amino acid residues, namely, His-63, His-119, Asp-209, His-213 are responsible for manganese coordination. A conserved residue of Tyr-35 is responsible for the second coordination sphere of the metal [26]. A highly conserved Mn-SOD signature sequence with the pattern D-x-[WF]-E-H-[STA]-[FY] existed in Ps-Mn-SOD (DVWEHAYY). The predicted secondary structure contained 13 α-helices and 4 β-strands. The deduced theoretical isoelectric point was 5.05, and the molecular weight was 29.29 kDa. The instability index of 36.97 classified the protein as stable. The 3D model of Ps-Mn-SOD was predicted using the x-ray template of *Bacillus subtilis,* which shared 45.27% sequence identity (PDB ID: 2RCV) [27]. This model shows that Ps-Mn-SOD is presented as a homodimer, and each subunit embraces one manganese ion. The global and per-residue model qualities were assessed using the QMEAN scoring function [28]. GMQE and QMEAN4 Z-scores reached 0.64 and −2.63, respectively, suggesting the accuracy of predicted 3D model of Ps-Mn-SOD. Figure 1 and Supplementary Figure S1 provide the related structural information of Ps-Mn-SOD.

### 2.2. Homology and Phylogenetic Analysis

**Figure 2:**
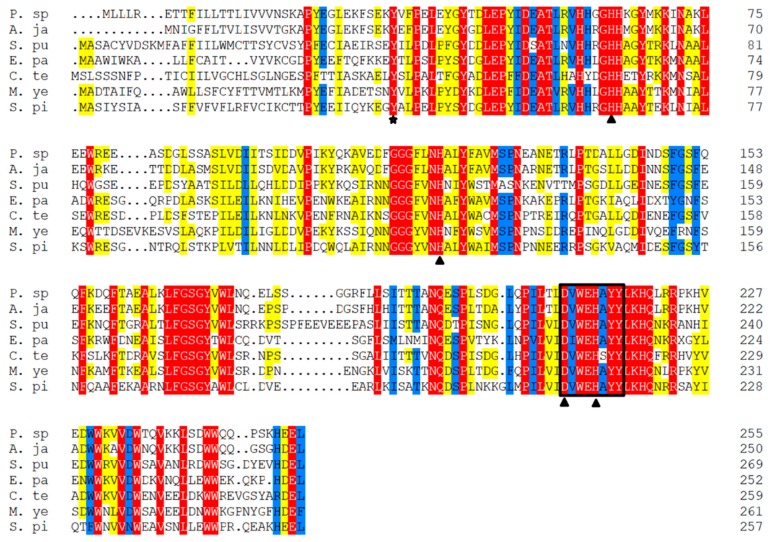
Multiple alignment of Ps-Mn-SOD with other invertebrates. Mn-SOD signature sequence is boxed. Triangles point to the active sites for manganese coordination. Asterisk points to the highly conserved Tyr-35 residue.

**Figure 3:**
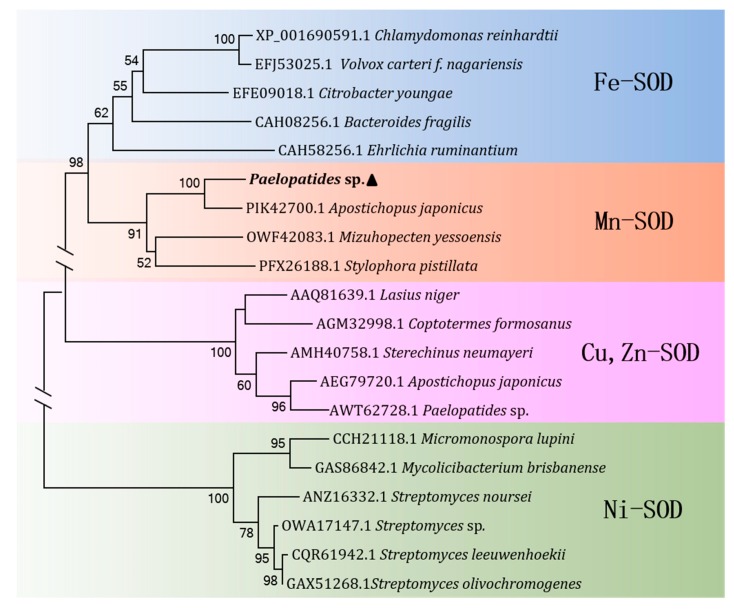
Neighbor-joining phylogenetic tree of SODs based on amino acid sequence homology. Bootstrap values below 50 are cut off. Ps-Mn-SOD is displayed in bold.

Multiple alignment and pairwise homology analysis between Ps-Mn-SOD and other invertebrates were performed, and the results are shown in Figure 2 and Supplementary Table S1. Multiple alignment of Ps-Mn-SOD with other invertebrates indicated that four amino acids were responsible for manganese binding, and the signature sequences are highly conserved in different Mn-SOD sources and were also identified in Ps-Mn-SOD (Figure 2). The highest similarity and identity were shared with *Apostichopus japonicus* (83.9% and 78.0%), followed by *Capitella teleta* (66.9% and 47.9%), *Exaiptasia pallida* (66.3% and 47.7%), *Strongylocentrotus* *purpuratus* (65.1% and 47.0%), *Mizuhopecten yessoensis* (64.4% and 46.7%), and *Stylophora pistillata* (63.1% and 45.8%). To determine the type of SOD present, we performed phylogenetic analysis based on the amino acid sequences of the determined SOD types in Genebank (Figure 3). The results showed that the present SOD clustered with *A. japonicus* and evidently a Mn-SOD type with high bootstrap values.

### 2.3. Expression, Purification, and Validation of Ps-Mn-SOD

The Ps-Mn-SOD gene was expressed with a His-tag in *E. coli*. Supplementary Figure S2 shows the SDS-PAGE analysis results. Recombinant Ps-Mn-SOD was expressed under 0.1 mM IPTG at 15 °C for 24 h and produced a distinct band at approximately 30 kDa, consistent with the previously estimated molecular weight (Supplementary Figure S2, lanes 1 and 2). The protein was purified under native conditions due to its highly soluble expression in the supernatant (Supplementary Figure S2, lanes 3 and 4). The maximum protein yield approximated 4.39 mg/L culture. Western blot analysis was performed to verify its successful expression (Supplementary Figure S2, lanes 5 and 6).

### 2.4. Characterizations of Ps-Mn-SOD

#### 2.4.1. Effects of Temperature on Ps-Mn-SOD

**Figure 4:**
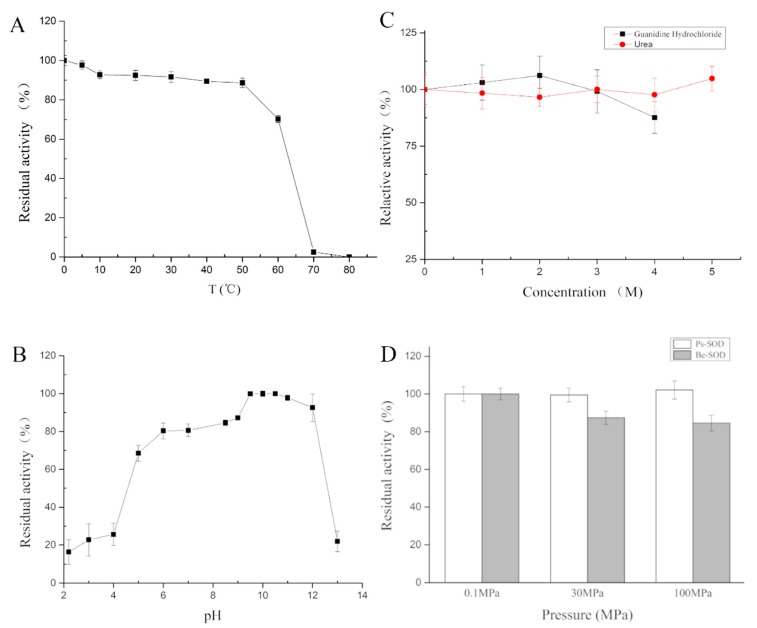
Effects of temperature (**A**), pH (**B**), urea and guanidine hydrochloride (**C**), and high hydrostatic pressure (**D**) on Ps-Mn-SOD. Ps-SOD and Be-SOD represent SOD from *Paelopatides* sp. and bovine erythrocytes, respectively.

The activity of Ps-Mn-SOD was determined from 0 °C to 80 °C, with the optimum temperature observed at 0 °C. A stable activity was observed at low temperatures, with > 70% activity highlighted from 0 °C to 60 °C. The activity was maintained at 2.53% at 70 °C and lost at 80 °C (Figure 4A).

#### 2.4.2. Effects of pH on Ps-Mn-SOD

The activity of recombinant Ps-Mn-SOD was measured under pH 2.2–13.0, with an optimum pH observed at 10.5 (Figure 4B). Ps-Mn-SOD could resist extreme pH values (> 20% at pH 3.0–13.0) and showed optimal activity (> 70%) at pH 5.0–12.0.

#### 2.4.3. Effects of Chemicals on Ps-Mn-SOD

The effects of metal ions on Ps-Mn-SOD activity were determined at 0.1 or 1 mM final concentration (Table 1). Ps-Mn-SOD activity was inhibited by Mn^2+^, Co^2+^, Ni^2+^, Zn^2+^, and 1 mM Cu^2+^ and Ba^2+^. In particular, Co^2+^ showed more significant inhibition effect on Ps-Mn-SOD activity. Mg^2+^ and Ca^2+^ showed minimal effects.

Table 2 provides the effects of inhibitors, detergents, and denaturants on Ps-Mn-SOD activity. Ps-Mn-SOD activity was strongly inhibited by ethylene diamine tetraacetic acid (EDTA) and SDS and especially sensitive to SDS. Reductant dithiothreitol (DTT) and β-mercaptoethanol (β-ME) minimally affected enzyme activity. Detergents of Tween 20, Triton X-100, and Chaps slightly enhanced enzyme activity at 0.1% concentration.

The enzyme could resist the strong denaturation of urea and guanidine hydrochloride (Figure 4C) and maintain an almost full activity after 1 h treatment in 5 M urea or 4 M guanidine hydrochloride.

**Figure 5:**
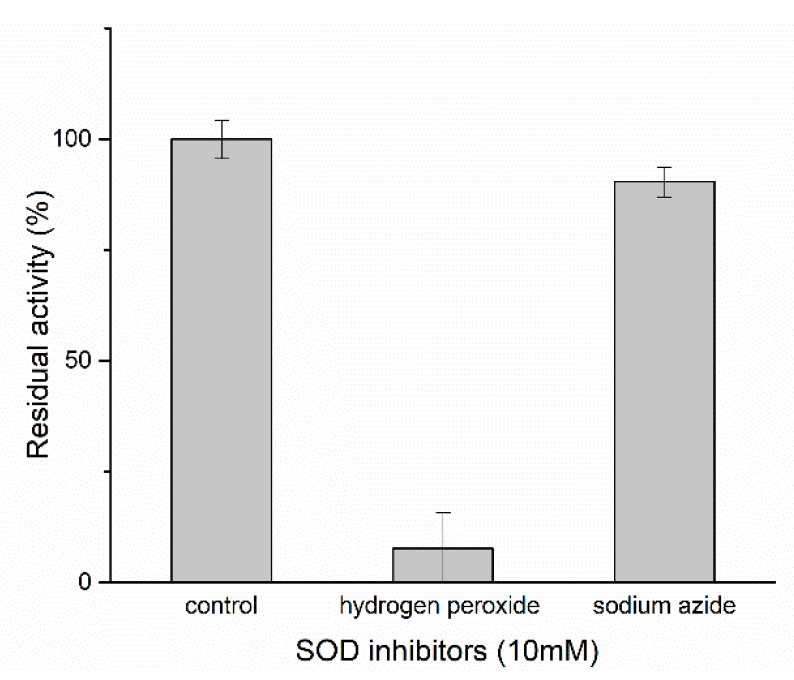
SOD type assay.

Hydrogen peroxide and sodium azide were used to determine the SOD type (Figure 5 and Supplementary Figure S4). After treatment of the recombinant Ps-Mn-SOD using 10 mM hydrogen peroxide and sodium azide at 25 °C for 1 h, the relative activities were 7.73% and 90.39%, respectively. This showed that the SOD from *Paelopatides* sp. belongs to Fe/Mn-SOD family, in accordance with previous phylogenetic analysis and 3D structure prediction.

#### 2.4.4. Effects of Digestive Enzymes on Ps-Mn-SOD

Digestion experiment was performed to test the stability of recombinant Ps-Mn-SOD in digestive fluid. Residual enzyme activity was measured after different incubation times for 0–4 h at 37 °C and pH 7.4. As shown in Table 3 and Supplementary Table S2, although the Ps-Mn-SOD sequence putatively contains 30 chymotrypsin and 23 trypsin cleavage sites, the enzyme could still maintain intact activity after 4 h treatment at an enzyme/substrate (*w*/*w*) ratio of 1/100.

#### 2.4.5. Effects of High Hydrostatic Pressure on Ps-Mn-SOD

As shown in Figure 4D, the recombinant Ps-Mn-SOD could maintain full activity with increasing hydrostatic pressure until 100 MPa. By contrast, the SOD from bovine erythrocytes exhibited reduced activity of 84.57% when the pressure reached 100 MPa.

#### 2.4.6. Kinetic Parameters

The kinetic parameters of recombinant Ps-Mn-SOD were determined using a series of xanthine (0.006–0.6 mM) concentrations at 37 °C and pH 8.2 (Supplementary Figure S3) based on the Michaelis–Menten equation. The Km and Vmax values of Ps-Mn-SOD were 0.0329 ± 0.0040 mM and 9112 ± 248 U/mg, respectively. The R^2^ value of the curve fitting was 0.9815.

## 3. Discussion

Mn-SODs are predominantly found in mitochondria, as the first line of antioxidant defense, which are involved in cellular physiology, such as cell impairment and immune-responsive [8]. The important biological functions of Mn-SODs have attracted increasing attention among researchers. Novel Mn-SODs with remarkable characteristics will have great applications in food, cosmetic, and pharmaceutical industries. In the present study, a novel and kinetically stable Mn-SOD derived from hadal sea cucumber was cloned, expressed, and characterized.

Based on preliminary data, the Ps-Mn-SOD is frigostabile, consistent with the fact that the protein was derived from hadal area, which maintained > 90% activity below 20 °C with the optimum temperature observed at 0 °C. In contrast, Mn-SOD from ark shell, *Scapharca broughtonii*, showed <40% activity below 20 °C [4]. Mn-SOD from seahorse, *Hippocampus abdominalis*, showed <80% and continuously reduced activity below 20 °C [8].

Mn-SODs in several sources have been found to function at wide pH values. For example, a hyperthermostable Mn-SOD from *Thermus thermophilus* HB27 maintained >70% activity at pH 4.0–8.0 [29]; Mn-SOD from deep-sea thermophile *Geobacillus* sp. EPT3 maintained >70% activity at pH 7.0–9.0 [30]; and Mn-SOD from *Thermoascus aurantiacus* var. *levisporus* only maintained >40% activity at pH 6.0–9.0 [31]. In contrast, the present Ps-Mn-SOD could maintain >70% activity at pH 5.0–12.0, showing remarkably wide pH values adaptation. Furthermore, after 1 h treatment in extremely acidic (pH 2.2) or alkaline (pH 13.0) conditions, Ps-Mn-SOD still maintained ~20% activity, showing remarkable stability to extreme pH values. The pH assays also showed that Ps-Mn-SOD is more stable under alkaline (pH 8.5–12.0) than acidic (pH 2.2–5.0) conditions. Metal ligands may undergo protonation at low pH but exhibit stability in alkaline conditions [32]. Similar studies on seahorse and bay scallop SODs were also reported [8,33].

Ps-Mn-SOD is relatively stable in chemicals, such as urea, guanidine hydrochloride, β-ME, DTT, etc. It maintained almost 100% activity after 1 h treatment of 5 M urea or 4 M guanidine hydrochloride at 25 °C, showing excellent resistance to strong protein denaturants. By comparison, the Mn-SOD from deep-sea thermophile *Geobacillus* sp. EPT3 maintained > 70% residual activity in 2.5 M urea or guanidine hydrochloride after 30 min treatment [30]. Fe-SOD from Antarctic yeast *Rhodotorula mucilaginosa* showed relatively low tolerance to urea [34]. However, based on our obtained data (unpublished and [35]), SODs from hadal sea cucumbers constantly exhibited excellent resistance to perturbation of denaturants. In addition, Ps-Mn-SOD maintained 97.00% and 99.22% residual activity after 1 h treatment of 10 mM DTT and 1% Triton X-100, respectively. While Mn-SOD from deep-sea thermophile *Geobacillus* sp. EPT3 only maintained 84.10% and 70.30% activity after 30 min treatment of corresponding chemicals [30].

As expected, Ps-Mn-SOD could also resist the perturbation by high hydrostatic pressure compared to the homolog from atmospheric pressure organism, because it was derived from a hadal field. Given the limitations of our equipment, the experiment was not performed at pressure more than 100 MPa. In fact, Ps-Mn-SOD might resist >100 MPa hydrostatic pressure. Similar results have been reported in other deep-sea enzymes, such as RNA polymerase from *Shewanella violacea* [36], N-acetylneuraminate lyase from *Mycoplasma* sp. [37], and lactate dehydrogenase b from *Corphaenoides armatus* [38]. Nonetheless, the sensitivity of enzymes to high hydrostatic pressure is not always related to the depth where the organisms lived. For example, two polygalacturonases from the hadal yeast *Cryptococcus liquefaciens* strain N6 exhibited an almost constant activity from 0.1 to 100 MPa. While, at the same pressure, polygalacturonase from *Aspergillus japonicus,* which lives under atmospheric pressure, increased by approximately 50% [39]. However, limited studies reported in detail the pressure assays of SODs, proving the difficulty in the interpretation of their pressure tolerance mechanism.

Altogether, these features render Ps-Mn-SOD a potential candidate in the biopharmaceutical and nutraceutical fields.

## 4. Materials and Methods

### 4.1. Material and Reagents

Hadal sea cucumber was collected at the depth of 6500 m in the Mariana Trench (10° 57.1693′ N 141° 56.1719′ E). Total RNA was extracted using RNeasy Plus Universal Kits from Qiagen, Hilden, Germany, and reverse-transcribed to cDNA. The transcriptome was obtained by sequencing assembly and annotation by Novogene Company (Tianjin, China). The following reagents were purchased from Takara, Tokyo, Japan: PrimeScript^TM^ II 1st strand cDNA Synthesis Kit, PrimeSTAR^®^ GXL DNA Polymerase, *E. coli* DH5α, and pG-KJE8/BL21 competent cells, pCold II vector, restriction enzymes *BamH I,* and *Pst I*, T4-DNA ligase, and DNA and protein markers. The 1 mL Ni-NTA affinity column, BCA protein assay kit, primers, and trypsin/chymotrypsin complex (2400:400) were obtained from Sangon Biotech Company, Shanghai, China. Polyvinylidene difluoride (PVDF) membrane was obtained from Millipore Company, USA. The primary (ab18184) and secondary antibodies (ab6789) were obtained from Abcam, Cambridge, UK. Pierce™ ECL Plus Western blot analysis substrate was obtained from ThermoFisher, Waltham, MA, USA.

### 4.2. Cloning and Recombinant

For the manganese SOD (Ps-Mn-SOD) gene, the Mn-SOD sequences of Holothuroidea in GenBank were submitted to the transcriptome database of *Paelopatides* sp. to run a local blast using Bioedit 7.0 software. The open reading frame (ORF) of Ps-Mn-SOD (deleted signal peptide) was amplified by primers Ps-Mn-SOD-S: CGGGATCCAAGGCTCCGTATGAAGGCCTGGAGA and Ps-Mn-SOD-A: AACTGCAGTCACAATTCTTCATGTTTAGATGGC using the cDNA as template (the underlined restriction enzyme sites). The sequence was submitted to GenBank database with accession numbers MK182093. The purified and digested PCR product was ligated with pCold II vector. The recombinant plasmids, that is, pCold II-Ps-Mn-SOD, were transformed into *E. coli* DH5α, and positive clones were verified by sequencing.

### 4.3. Protein Overproduction, Purification, and Confirmation

The recombinant plasmids were transformed into *E. coli* chaperone competent cells pG-KJE8/BL21, which were inoculated in liquid Luria-Bertani medium (containing 100 μg/mL ampicillin, 20 μg/mL chloramphenicol, 0.5 mg/mL L-arabinose, and 2 ng/mL tetracycline), proliferated at 37 °C until the OD_600_ reached 0.4–0.6, cooled on an ice–water mixture for 40 min, added isopropyl β-D-1-thiogalactopyranoside (IPTG) with a final concentration of 0.1 mM, and then incubated for 24 h at 15 °C to produce the recombinant protein. Cells were harvested, washed with **1 ×** phosphate-buffered saline, resuspended in binding buffer (50 mM Na_3_PO_4_, 300 mM NaCl, and 20 mM imidazole, pH 7.4), and then sonicated on ice. The supernatant harboring the recombinant protein was separated from cell debris by centrifugation at 12000 g and 4 °C for 20 min and then applied to 1 mL Ni-NTA column for purification of the target protein based on its 6× His-tag, according to the manufacturer’s instructions. The harvested target protein was dialyzed with 1 × tris buffered saline (TBS) at 4 °C for 24 h against three changes of 1 × TBS and finally stored at −80 °C for further experiments. The expression condition was analyzed on 12% sodium dodecyl sulfate polyacrylamide gel electrophoresis (SDS-PAGE) and confirmed using Western blot analysis. The recombinant protein on 12% SDS-PAGE gel was transferred to a PVDF membrane, which was successively incubated with primary (diluted 1:5000) and secondary antibodies (diluted 1:10000), dyed with Pierce™ ECL Plus Western blot analysis substrate, and detected under chemiluminescent imaging system. Additional details were as described by Li et al. [35].

### 4.4. Bioinformatics Analyses

The amino acid sequence of Ps-Mn-SOD was translated using ExPASy translation tool (http://web.expasy.org/translate/). The signal peptide, secondary structure, motif sequences, and 3D homology model were predicted by SignalP 4.1 Server (http://www.cbs.dtu.dk/services/SignalP/), Scratch Protein Predictor (http://scratch.proteomics.ics.uci.edu/), InterPro Scan (http://www.ebi.ac.uk/InterProScan/), and Swiss model server (http://swissmodel. expasy.org/) [40], respectively. The physicochemical properties of Ps-Mn-SOD were predicted using ExPASy ProtParam tool (http://web.expasy.org/protparam/). The possible cleavage sites of trypsin and chymotrypsin on Ps-Mn-SOD were predicted using the peptide cutter software (http://web.expasy.org/peptide_cutter/). Multiple alignments of Ps-Mn-SOD were processed using DNAMAN 7.0.2 software. Homology analysis was constructed by pairwise alignment tool (https://www.ebi.ac.uk/Tools/psa/emboss_needle/). The neighbor-joining phylogenic tree was generated in MEGA 7.0 with bootstrap values 1000.

### 4.5. Enzyme Assays

SOD activity was determined via spectrophotometric method using the SOD assay kit from Nanjing Jiancheng Institute of Biology and Engineering (Code No. A001-1-1, Nanjing, China). Each measurement point contained three replicates, and the results are shown as mean (n = 3) ± standard deviation (SD). The 1 × TBS was used as the blank control. One unit of SOD activity was defined as the amount of enzyme that inhibited 50% of chromogen production at 550 nm.

The purified Ps-Mn-SOD was quantified, and residual activities were determined after incubation under different variables, including temperature, pH, chemicals, digestive enzymes, and high hydrostatic pressure. Considering temperature, proteins were treated from 0 °C to 80 °C for 15 min with an interval of 10 °C [33,34]. Proteins were treated at pH 2.2–13 for 1 h at 25 °C [4,34]. The enzymatic activity at optimum temperature and pH was set as 100%. With regard to chemicals, the proteins were mixed with an equal treatment solution at different final concentrations for 40 min at 25 °C [34,41]. The incubation time of urea, guanidine hydrochloride, hydrogen peroxide, and sodium azide was expanded to 1 h. The enzyme activity without chemicals was set as 100%. For proteolytic susceptibility assay, the mass ratio of recombinant Ps-Mn-SOD and trypsin/chymotrypsin complex was 1:100, and the group incubated for 0 h was considered with 100% enzyme activity [34,42]. For high hydrostatic pressure, proteins were treated at 0.1, 30, and 100 MPa for 2 h at 5 °C. The enzyme activity at 0.1 MPa was set as 100%, and bovine erythrocyte SOD was selected for comparison from atmospheric organism. Kinetics of Ps-Mn-SOD were measured as previously described by Li et al. [35].

### 4.6. Statistical Analysis

Independent sample *T*-test was used for statistical analysis for each of the two groups using SPSS 21.0 (IBM Company, Armonk, NY, USA); *p* < 0.05 was considered statistically significant.

## Figures and Tables

**Table 1:** Effects of metal ions on Ps-Mn-SOD. ** *p* < 0.01.

Divalent Metal Ions	Concentration/mmol·L^−1^	Relative Activity/%
Control	—	100 ± 2.39
Mn^2+^	0.1	92.89 ± 1.53 **
	1	84.99 ± 2.77 **
Co^2+^	0.1	80.13 ± 1.23 **
	1	61.49 ± 1.54 **
Ni^2+^	0.1	95.70 ± 2.38 **
	1	94.42 ± 2.92 **
Zn^2+^	0.1	90.25 ± 1.76 **
	1	90.99 ± 4.63 **
Cu^2+^	0.1	98.39 ± 3.97
	1	88.99 ± 5.44 **
Ba^2+^	0.1	99.16 ± 2.18
	1	95.94 ± 2.40 **
Mg^2+^	0.1	100.68 ± 3.27
	1	100.61 ± 2.16
Ca^2+^	0.1	99.71 ± 1.13
	1	100.39 ± 4.48

**Table 2:** Effects of inhibitors, reductant, and detergents. * *p* < 0.05; ** *p* < 0.01.

Divalent Metal Ions	Concentration	Relative Activity/%
Control	—	100 ± 2.84
EDTA	1 mmol·L^−1^	64.33 ± 3.08 **
	10 mmol·L^−1^	58.03 ± 2.59 **
DTT	1 mmol·L^−1^	96.36 ± 4.65
	10 mmol·L^−1^	97.00 ± 5.46
β-ME	1 mmol·L^−1^	96.53 ± 4.47
	10 mmol·L^−1^	101.85 ± 3.72
Tween 20	0.1%	109.96 ± 6.62 **
	1%	105.01 ± 3.28 **
Chaps	0.1%	103.13 ± 2.32 *
	1%	99.32 ± 3.66
Triton X-100	0.1%	105.02 ± 3.2 9**
	1%	99.22 ± 3.79
SDS	0.1%	5.21 ± 3.45 **
	1%	6.11 ± 4.15 **

**Table 3:** Cleavage effect of digestive enzyme on Ps-Mn-SOD at different time periods. Results are shown as mean (*n* = 3) ± SD. ** *p* < 0.01.

Time (h)	Relative Activity (%)
0	100 ± 2.21
1	108.66 ± 5.70
2	104.46 ± 4.54
3	103.73 ± 3.24
4	106.72 ± 4.80

## Supplementary Materials

The following are available online at http://www.mdpi.com/1660-3397/17/2/84/s1, Figure S1: The predicted 3D model of Ps-Mn-SOD. Red spheres represent manganese ions. (**A**) homodimer. (**B**) close-up of the manganese ion binding site. Figure S2: Analysis of SDS-PAGE. M: protein marker, Lane 1: total proteins before induction, Lane 2: total proteins after induction, Lane 3: inclusion body after ultrasonication, Lane 4: supernatant after ultrasonication, Lane 5: Western blot of recombinant protein, Lane 6: purified protein. Figure S3: The curve of kinetic parameters of Ps-Mn-SOD. Figure S4: SOD type assay. The result is expressed using specific activity. Table S1. Pairwise alignment analysis between Ps-Mn-SOD and other species. Table S2. The prediction of cleavage site of Ps-Mn-SOD.
