# Supplementary material for: Characteristics of a Novel Manganese Superoxide Dismutase of a Hadal Sea Cucumber (Paelopatides sp.) from the Mariana Trench
Source: Mar Drugs. 2019 Feb 1;17(2):84. doi: 10.3390/md17020084 (PMC6410416; doi:10.3390/md17020084)
Supplement: Supplementary file 1 [file marinedrugs-17-00084-s001.pdf]

marine drugs

Supplementary Materials

Characteristics of a Novel Manganese Superoxide Dismutase of a Hadal Sea Cucumber (*Paelopatides* sp.) from the Mariana Trench

Yanan Li [1,2], Xue Kong [1,2] and Haibin Zhang [1,*]

[1] Institute of Deep-Sea Science and Engineering, Chinese Academy of Sciences, Sanya 572000, China; liyn@idsse.ac.cn (Y.L.); kongx@sidsse.ac.cn (X.K.)
[2] College of Earth and Planetary Sciences, University of Chinese Academy of Sciences, Beijing 100039, China
* Correspondence: hzhang@idsse.ac.cn; Tel.: +86-0898-8838-0935; Fax: +86-0898-8838-0935

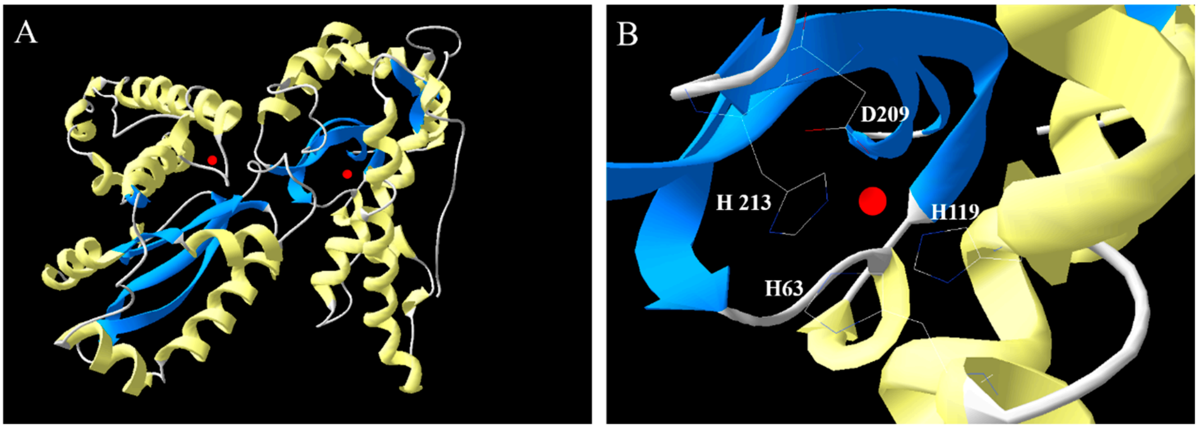

Figure S1. The predicted 3D model of Ps-Mn-SOD. Red spheres represent manganese ions. (**A**) homodimer. (**B**) close-up of the manganese ion binding site.

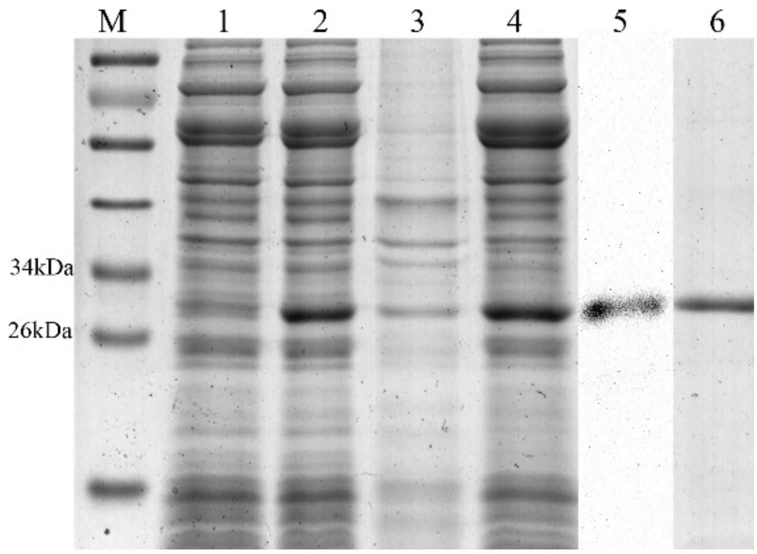

Figure S2. Analysis of SDS-PAGE. M: protein marker, Lane 1: total proteins before induction, Lane 2: total proteins after induction, Lane 3: inclusion body after ultrasonication, Lane 4: supernatant after ultrasonication, Lane 5: Western blot of recombinant protein, Lane 6: purified protein.

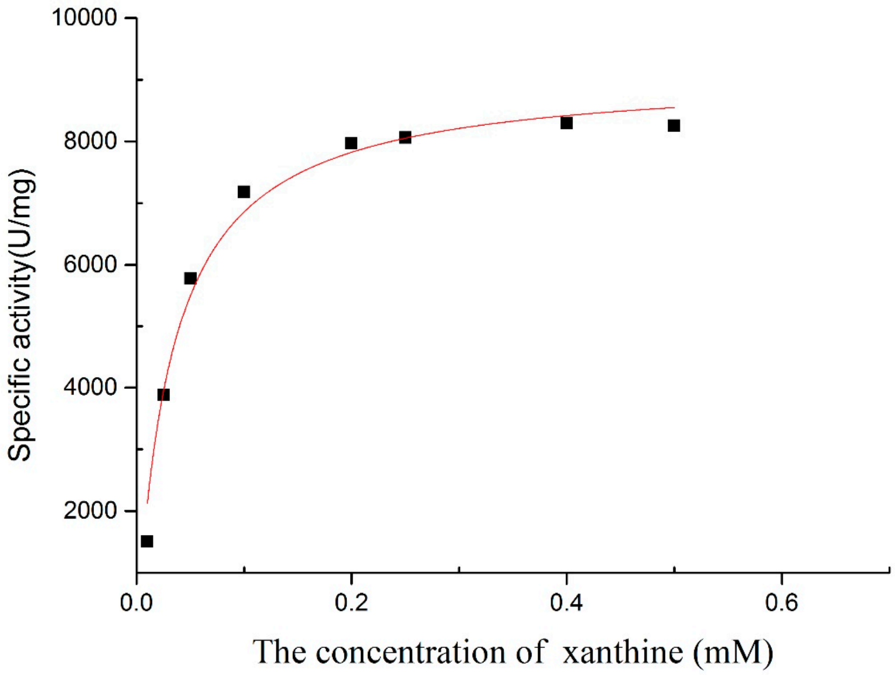

Figure S3. The curve of kinetic parameters of Ps-Mn-SOD.

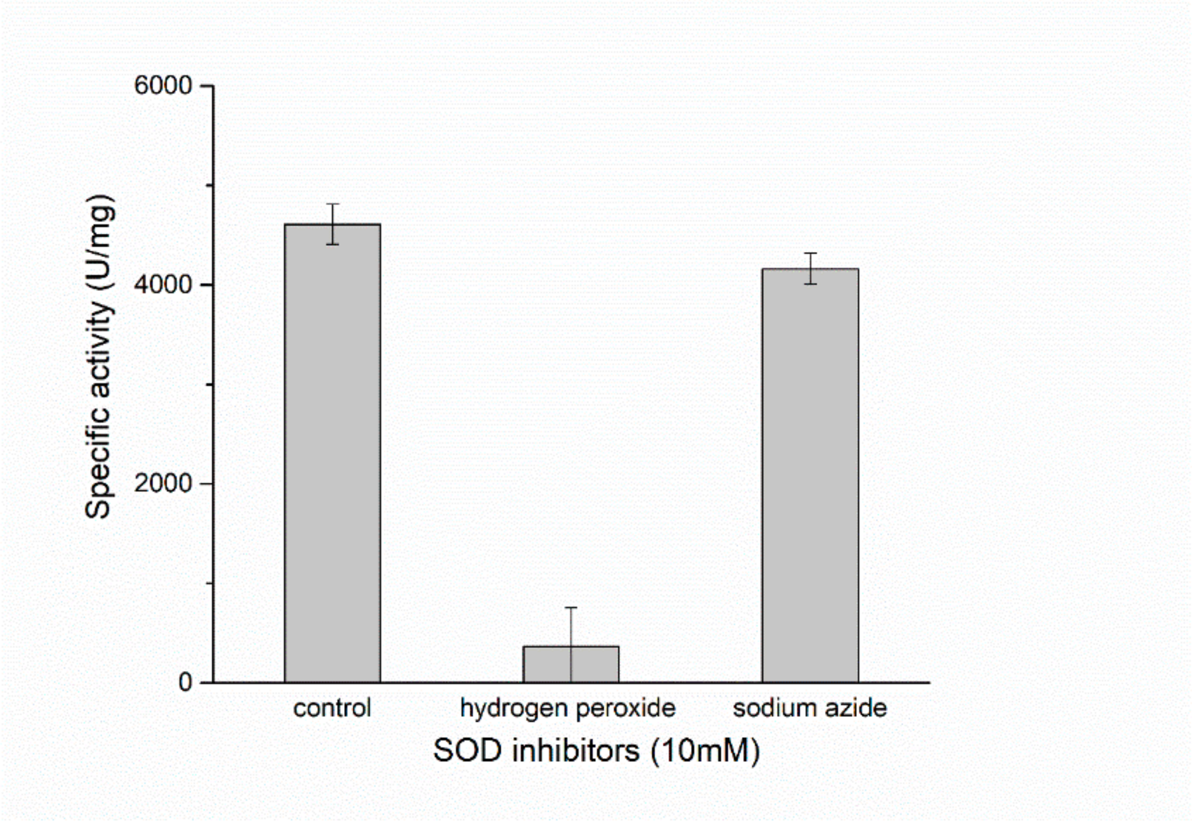

Figure S4. SOD type assay. The result is expressed using specific activity.

Table S1. Pairwise alignment analysis between Ps-Mn-SOD and other species.

Species	Genbank No.	Abbreviation	Size (aa)	Similarity%	Identity%	Gap%
Apostichopus japonicus	PIK42700.1	A. ja	250	83.9	78.0	2.0
Strongylocentrotus purpuratus	XP_791826.1	S. pu	269	65.1	47.0	13.5
Exaiptasia pallida	KXJ16556.1	E. pa	252	66.3	47.7	3.9
Capitella teleta	ELT98943.1	C. te	259	66.9	47.9	4.6
Mizuhopecten yessoensis	OWF42083.1	M. ye	261	64.4	46.7	8.9
Stylophora pistillata	PFX26188.1	S. pi	257	63.1	45.8	3.1

Table S2. The prediction of cleavage site of Ps-Mn-SOD.

Name of Enzyme	No. of Cleavages	Positions of Cleavage Sites
Chymotrypsin-high specificity (C-term to [FYW], not before P)	30	9 25 31 35 42 44 50 67 78 105 112 116 122 123 149 152 155 159 167 171 173 184 211 215 216 230 231 236 245 246
Trypsin	23	5 22 30 34 57 65 69 70 74 79 104 107 135 156 165 183 218 222 225 232 240 241 251
